# Case report: Cases of narcolepsy misdiagnosed as other psychiatric disorders

**DOI:** 10.3389/fpsyt.2022.942839

**Published:** 2022-11-03

**Authors:** Zhongxia Shen, Yibin Shuai, Shaoqi Mou, Yue Shen, Xinhua Shen, Shengliang Yang

**Affiliations:** ^1^School of Medicine, Southeast University, Nanjing, China; ^2^Sleep Medical Center, Huzhou Third Municipal Hospital, The Affiliated Hospital of Huzhou University, Huzhou, China; ^3^Department of Psychiatry, Wenzhou Medical University, Wenzhou, China

**Keywords:** narcolepsy, misdiagnosis, psychiatric disorder, case report, excessive daytime sleepiness (EDS)

## Abstract

Narcolepsy is characterized by uncontrollable excessive daytime sleepiness, paroxysmal cataplexy, sleep paralysis, and hallucinations. It is often misdiagnosed as psychiatric disorders such as depression and schizophrenia, resulting from the overlap in symptoms and a lack of understanding of narcolepsy. In the present study, three cases of narcolepsy misdiagnosed as depression, dissociative disorder, and schizophrenia are presented to emphasize the high occurrence of the misdiagnosis of narcolepsy in clinical practice. The main reasons for this dilemma are attributed to the lack of adequate sleep, medicine, education, as well as specialized professional technicians. A multi-disciplinary team composed of psychiatrists and sleep specialists should be established to deal with this problem.

## Introduction

Narcolepsy is a lifelong sleep disorder characterized by intermittent arousal in rapid eye movement (REM) sleep with the symptoms of excessive daytime sleepiness (EDS), sleep fragmentation, sleep-related hallucinations, sleep paralysis, and cataplexy (1, 2); patients with narcolepsy also share the symptoms of eating disorders, affective symptoms, and anxiety. These symptoms are not confined to just one cluster but span several domains with high comorbidity psychiatric disorders such as depression, anxiety, and schizophrenia (2). Narcolepsy is often misdiagnosed as other psychiatric disorders resulting from the overlap in symptoms and a lack of understanding of sleep disorders (2, 3). There is still little understanding of narcolepsy in clinical practice; misdiagnosis and missed diagnosis are common, and the peak period of onset is in childhood (2). Therefore, correct understanding, early diagnosis, and reasonable treatment can significantly improve growth, development, and quality of life. In this study, we introduced three cases of narcolepsy that were misdiagnosed as psychiatric disorders in the early stages of life and explored the potential reasons for misdiagnosis.

## Case presentation

### Case 1

#### Chief complaints

A 17-year-old woman visited a psychiatry outpatient service with EDS and depression for 6 years.

#### History of present illness

The patient gradually developed EDS, inattention in class, and began sleeping in class about 6 years ago without any obvious inducement. Her parents thought she was too tired to study and asked her to go to bed before 21:00 every day, but more than 8 h of sound sleep at night could not make her perform better during the day. Meanwhile, she ate more and had significant weight gain. Because of continued EDS, the patient was unable to study, which led to a decline in academic performance. She would often attempt to keep herself awake but fall asleep even while studying, chatting, and eating. Over the next 6 years, the symptoms continued to aggravate. These symptoms led to significant socio-occupational dysfunction, and she became distressed. She visited multiple physicians, including neurologists and psychiatrists. After repeated examination of thyroid function, electroencephalography (EEG), computed tomography, and cerebrospinal fluid analysis, viral encephalitis and other nervous system diseases, such as epilepsy, were excluded. Her family thought that she might have psychological problems, so she was brought to the psychological department of our hospital for treatment. She was diagnosed with “Major Depressive Disorder” and was treated with sertraline (100 mg qd) but showed poor improvement. After entering high school, the patient again experienced the same pattern of sleepiness. As a result, she was so distressed that she lost interest in everyday life. After a comprehensive physical examination and supplementary examination, such as repeated EEG and head magnetic resonance imaging, the diagnosis of “depression” was confirmed. However, the effect of antidepressant treatment (including venlafaxine 150 mg qd and bupropion 150 mg qd) was not satisfactory.

#### History of past illness and family history

The patient had no major physical and mental illnesses in the past. There was no positive family history of any sleep disorders or mental disorders.

#### Diagnostic assessment and treatment

The patient was hospitalized in the sleep medical center. Tests for liver function, renal function, serum electrolytes, thyroid function, and dynamic EEG revealed no abnormalities. After multiple sleep latency test (MSLT) examinations, a diagnosis of “narcolepsy” was considered. Overnight polysomnography (PSG) in our sleep medical center indicated that the total sleep time (TST) was 7:42:0, sleep efficiency (SE; TST/time in bed [TIB]) was 90%, the N3 duration was 93 min, and the REM phase duration was 94.5 min; obstructive sleep apnea was excluded. Repeated MSLT examination during five naps showed three REM episodes and three sleep-onset REM periods (SOREMPs), the mean sleep latency was 7.1 min, and the mean sleep latency of REM was 8.8 min during daytime. Combined with a comprehensive mental examination, according to the International Classification of Disease 10th Revision (ICD-10), she was diagnosed with narcolepsy (G47.4); thus, antidepressants were stopped, and modafinil (100 mg qd) was prescribed to treat the narcolepsy.

#### Outcome and follow-up

One week after the treatment, the symptoms of EDS disappeared, with continuous improvement in academic performance after modafinil treatment; depressive symptoms also subsided, with the caveat that the patient will still have EDS symptoms if she stopped meds over the weekends. So far, she continues treatment with modafinil (100 mg qd) with no obvious side effects.

### Case 2

#### Chief complaints

A 14-year-old female student was hospitalized due to ‘having “paroxysmal cataplexy” for more than 2 years, with her condition being aggravated during the past 6 months'.

#### History of present illness

The patient had an acute onset of illness after being frightened for more than 2 years ago. She had sudden dizziness with no change in body position for several days. Her head felt heavy and she had visual rotation, but there was no nausea and vomiting, chest tightness, or fever. Since then, the patient's sleep had been irregular, and day and night sleep patterns were reversed. In the beginning, the patient was treated in a local hospital. EEG and transcranial Doppler ultrasound showed mild abnormalities, and no noticeable abnormalities were found during head CT. The patient went to the pediatric hospital, and no apparent abnormalities were found after relevant examinations. She was diagnosed with “dizziness” and was treated with traditional Chinese medicine (the specific details are unknown), without desired effects. The patient went to a psychiatric hospital and was diagnosed with “sleep-wake rhythm disorder, epilepsy.” After treatment with lorazepam (0.75 mg qn), fluoxetine (20 mg qd), olanzapine (5 mg qn), and topiramate (0.1 qd), intermittent dizziness persisted and, sometimes, there were occurrences of even “cataplexy” not accompanied by convulsions. Dynamic EEG was performed, but no epileptic waves were found. More than 1 year before this case presentation, the patient suffered from paroxysmal dizziness and fell slowly to the ground when she was in school but did not sustain a physical injury. When responders opened her eyes, they found that her eyes were turned up, her face and lips remained unchanged, and she had no limb convulsions. After 2–5 min, the patient was awake and lucid. The patient dropped out of school due to paroxysmal cataplexy. After quarreling with her mother, the episodes gradually increased in occurrence to 1–4 times/day, with the same manifestation. Therefore, the patient went to another pediatric hospital and was diagnosed with “cataplexy, dissociative disorder,” her original drug treatments were stopped, and she was switched to sertraline (50 mg qd). After treatment, her condition improved mildly, and episodes of cataplexy decreased significantly to a frequency of about 1–2 per month. Six months before this case presentation, the condition of the patient worsened for no apparent reason, with similar symptoms as in her prior presentation, but attacks were more frequent, at more than 10 times per day. Therefore, the patient went to the pediatric hospital again and resumed sertraline (50 mg qd), but the treatment was ineffective. She then went to another pediatric hospital and was diagnosed with “viral encephalitis” after a cerebrospinal fluid examination. Cataplexy still occurred frequently after antiviral treatment, so the patient was transferred to a hospital in Beijing. During hospitalization, cerebrospinal fluid was rechecked. No obvious abnormalities were found, so she was transferred to the department of psychology for further treatment.

#### History of past illness and family history

The patient had no major physical and mental illnesses in the past. Her parents (now divorced) were busy with work and her grandfather provided primary care. The patient had no positive family history of any sleep disorders or mental disorders in her family.

####  Diagnostic assessment and treatment

The patient experienced many laboratory examinations, and we only listed the following subset here. Transcranial Doppler ultrasound, video EEG, and evoked potential EEG showed no obvious abnormalities (July 2019; Children's Hospital of Fudan University). Head MRI showed that a deep choroidal cyst in the right temporal lobe was possible, and there was minor inflammation in the right posterior ethmoid sinus group; head MRA indicated that the posterior cerebral artery on the cephalic side was not displayed, and there were no obvious abnormal signs in the others; cerebrospinal fluid was found to be colorless and transparent, leukocytes were 29 × 10^6^/l, mainly multinucleated, with no obvious biochemical abnormalities and a negative culture (May 2021; Zhoushan Women's and Children's Hospital). The lying and sitting position test was negative; the dynamic electrocardiogram showed sinus rhythm, occasional ventricular premature contractions, intermittent ST-segment and T-wave changes, and a normal QT interval (20 September 2021; the First Hospital of Peking University).

Overnight PSG performed in our sleep medical center showed a TST of 8:13:0, an SE (TST/TIB) of 92%, an N3 duration of 105 min, and a REM duration of 90.0 min; obstructive sleep apnea was excluded. MSLT examination during five naps showed three REM episodes and three SOREMPs, a mean sleep latency of 3.7 min, and a mean sleep latency of REM of 4.7 min in the daytime. The diagnosis of the patient was corrected to “narcolepsy” after MLST examinations. Treatment with modafinil (100 mg qd) was prescribed to treat cataplexy syndrome, while treatment with sertraline (50 mg qd) continued. Zopiclone (7.5 mg qn) was prescribed to treat delayed sleepiness.

#### Outcome and follow-up

After 1 week of pharmaceutical treatment, the cataplexy syndrome disappeared and EDS improved, but the sleep rhythm disorder persisted with a presentation of late sleep and late waking up. The patient still cannot return to school. Her medication adherence is not very good, and she occasionally has cataplexy attacks when her mood changes, such as while quarreling with her mother.

### Case 3

#### Chief complaints

An 18-year-old woman presented to our department with a chief complaint of “Too much sleep, depression, and hallucination for 6 years.”

#### History of present illness

The patient began to gradually feel sleepier during the daytime, and she always felt fatigued during the daytime 6 years prior without any obvious inducement. The quieter the environment, the easier it was to fall asleep. Sometimes the patient even could fall asleep when walking. Due to her symptoms, the patient suffered a decline in academic performance and was misunderstood by her parents, causing emotional distress. She always felt exhausted and lost interest in almost everything except food. The patient experienced multimodal hallucinations while falling asleep. She would see an unknown person standing near her or terrible scenes that others could not see. She experienced the olfactory hallucination of the smell of honey and auditory hallucinations of her mother. She stated that sometimes she had the feeling that her “whole brain was filled with porridge”. She attributed this to some parts of her brain having been disconnected from others, and she stated that sometimes some of her thoughts were not hers. While experiencing these hallucinations, the patient could not move her limbs or speak; this phenomenon always happened before falling asleep or after coming out of sleep, daytime or night. Therefore, she often felt nervous and insecure and was afraid of being alone. She felt that the problem was out of her control and she had no future. After 3 months of symptom onset, she was diagnosed with “schizophrenia” in a local psychiatric hospital. She received treatment with sertraline and olanzapine during a 2-month hospitalization; symptoms slightly improved, but the details are obscure. The EDS symptoms of the patient became more serious and hallucinations persisted, and the patient could not continue her normal studies. Three years prior to the case presentation, she went to another psychiatric hospital in Shanghai for treatment. The diagnosis of schizophrenia was confirmed, and the patient successively received symptomatic treatment with olanzapine (5 mg qn) and aripiprazole (5 mg qd). The response to treatment was unsatisfactory, and the patient experienced worsened sleepiness. She became less confident and sometimes overate; the patient also presented with obesity. The patient denied that she had negative thoughts. Since the onset of her symptoms, she had no prominent periods of remission. Due to the impairment of social functions, she dropped out of school and could not perform her usual housework duties.

#### History of past illness and family history

The patient had been in good health history and denied that other members of the family had a history of mental disorders.

#### Diagnostic assessment and treatment

The patient was admitted to the sleep medical center of our hospital. Overnight PSG examination showed a TST of 7:38:16, SE (TST/TIB) of 93%, an N3 duration of 89 min, and a REM duration of 92.0 min; obstructive sleep apnea was excluded. MSLT examination during five naps reported five REM episodes and three SOREMPs; the mean sleep latency was 5.9 min, and the mean sleep latency of REM was 1.0 min during the daytime. Combined with a comprehensive mental examination, the patient was diagnosed with “narcolepsy” according to ICD-10 criteria. Her treatment was adjusted, treatment with aripiprazole was stopped, and bupropion (150 mg qd) was prescribed; after treatment for 1 week, the symptoms of hallucination subsided, but she still felt fatigued, had slow responses, and experienced occasional dizziness. The bupropion prescription (150 mg qd) was changed to modafinil (100 mg qd).

#### Outcome and follow-up

After 2 weeks of treatment with modafinil, EDS symptoms were significantly relieved; at the same time, hallucination symptoms completely disappeared, other manifestations were also significantly improved, and night sleep returned to normal. One month after being discharged from the hospital, the patient behaved normally and was able to perform her daily housework tasks.

## Discussion

Narcolepsy is frequently misdiagnosed as a psychiatric disorder in clinical practice, which occurred in the cases reported above, causing a delay in accurate diagnosis and treatment. Lee et al. (4) reported that more than 50% of patients with narcolepsy had been diagnosed with depression prior to narcolepsy (4). More than one-third of patients with narcolepsy have various forms of hallucinations even during daytime naps (5), which is easy to confuse with schizophrenia (2). Anxiety disorders, such as panic disorder, have been reported in as many as 53% of patients with narcolepsy (6). Furthermore, more than 23% of patients with narcolepsy had a comorbid clinical eating disorder (7). As concluded by John Khoury (1), an accurate diagnosis of narcolepsy will not be established until an average of 10 years after the onset of this disorder (1). Patients with narcolepsy display the symptoms of eating disorders, affective symptoms, and hallucinations (1).

Narcolepsy seriously damages the social functioning and quality of life of patients, which causes a high socioeconomic burden (8). The three patients reported herein were teenage students; EDS disabled their studies, and, if misdiagnosed as schizophrenia, the maintenance of anti-psychotics may cause a more difficult life if the diagnosis is not corrected in time. Peers, parents, teachers, patients themselves, and doctors often confused EDS with laziness or lack of motivation, which was reflected in these three reported cases. Meanwhile, substantial medical resources are used when the correct diagnosis is delayed, which was also reflected in these three cases. The main case characteristics of the three patients are listed in Table 1.

**Table 1:** Main characteristics of all three patients.

	**Symptoms**	**Results of MLT**	**SOREM**	**Treatment**	**Long-term evolution after treatment**
Case1	Excessive daytime sleepiness (EDS) and depression for 6 years.	3 REM episodes and 3 SOREMPs in 5 naps, the mean sleep latency was 7.1 min, the mean sleep latency of REM was 8.8 min in daytime.	3 SOREMPs in 5 naps.	Modafinil 100 mg qd.	The symptoms of EDS and depression disappeared with no obvious side effects.
Case2	Paroxysmal cataplexy for more than 2 years and aggravating for half a year.	3 REM episodes and 3 SOREMPs in 5 naps, the mean sleep latency was 3.7 min, the mean sleep latency of REM was 4.7 min in daytime.	3 SOREMPs in 5 naps.	Modafinil 100 mg qd, sertraline 50 mg qd, zopiclone 7.5 mg qn.	The syndrome of cataplexy disappeared and EDS relieved, but the sleep rhythm disorder still existed.
Case3	Too much sleep, depression, and hallucination for 6 years.	5 REM episodes and 3 SOREMPs in 5 naps, the mean sleep latency was 5.9 min, the mean sleep latency of REM was 1.0 min in daytime.	3 SOREMPs in 5 naps.	Bupropion 150 mg qd (stopped a week later), modafinil 100 mg qd.	The symptoms of EDS were significantly relieved, hallucination symptoms completely disappeared, retardation and other manifestations were also significantly improved, and night sleep returned to normal.

The diagnosis of narcolepsy must be confirmed by overnight PSG, followed by an MSLT. PSG is performed to ensure adequate nocturnal sleep and excludes other sleep disorders. To establish the diagnosis, the average MSLT sleep latency should be 8 min or less and patients should show REM episodes during at least two nap opportunities or in one nap opportunity if PSG of the previous night revealed the onset of REM sleep within 15 min of sleep onset (9, 10). Nowadays, only a few psychiatrists or psychologists in China received adequate education and training in sleep medicine for cases of suspected narcolepsy in patients. In fact, many psychiatric hospitals or departments in China have no PSG instruments. Even if the doctors are aware of the presence of narcolepsy, they have to consider the manifestation of psychiatric syndromes. Performing the MSLT requires professional technicians, as it is more than a simple PSG instrument. There are few hospitals that can test for narcolepsy-related biomarkers, such as hypocretin levels in cerebrospinal fluid (11).

A diagnosis of narcolepsy should be considered in atypical and refractory psychiatric illnesses, especially in children and adolescents. More importantly, psychiatrists and psychologists need further education in sleep medicine. Education plays a crucial role in the management of these patients. Considering the high comorbidity of narcolepsy and psychiatric illness, multi-disciplinary teams composed of psychiatrists and sleep specialists should be established to deal with this dilemma. Furthermore, in the three cases of narcolepsy reported above, all patients responded quickly to modafinil with good tolerance, as is reported by other studies (12). This suggested that it was not likely to be too late to administer proper medication for narcolepsy, and an optimistic prognosis can be expected with the maintenance of medication. Future studies should focus on exploring more practical approaches to identifying patients with narcolepsy and psychiatric illness.

## Data availability statement

The original contributions presented in the study are included in the article/supplementary material, further inquiries can be directed to the corresponding authors.

## Ethics statement

The studies involving human participants were reviewed and approved by the Ethics Committee of Huzhou Third Municipal Hospital. Written informed consent to participate in this study was provided by the participants' legal guardian/next of kin. Written informed consent was obtained from the minor(s)' legal guardian/next of kin for the publication of any potentially identifiable images or data included in this article.

## Author contributions

ZS, YShu, SM, YShe, and XS contributed to the treatment and information record of the case report. ZS and SY contributed to the medical treatment of the patient, conceptualization of the case report, and manuscript writing and editing. All authors have read and approved the final version.

## Funding

This study was partly supported by the Huzhou Public Welfare Research Project Social Development Category (2018GYB49, ZS) and the Social Development Project of Public Welfare Technology Application in Zhejiang Province in 2019 (LGF19H090002, ZS).

## Conflict of interest

The authors declare that the research was conducted in the absence of any commercial or financial relationships that could be construed as a potential conflict of interest.

## Publisher's note

All claims expressed in this article are solely those of the authors and do not necessarily represent those of their affiliated organizations, or those of the publisher, the editors and the reviewers. Any product that may be evaluated in this article, or claim that may be made by its manufacturer, is not guaranteed or endorsed by the publisher.
